# A midline posterior approach for treatment of os odontoideum via atlantoaxial reduction, bone graft fusion, and internal fixation

**DOI:** 10.3171/2020.1.FocusVid.19723

**Published:** 2020-01-01

**Authors:** Lei Zhao, Peng Wang, Weixin Li

**Affiliations:** Department of Neurosurgery, Tangdu Hospital, Air Force Medical University, Xi’an, Shaanxi, China

**Keywords:** os odontoideum, atlantoaxial reduction, bone graft fusion, internal fixation, video

## Abstract

Os odontoideum is a rare anomaly of the second cervical vertebra, which can result in the compression and injury of cervical spinal cord. This deformity is surgically challenging. The authors presented a case of a 50-year-old man with a 2-year history of numbness and weakness in four limbs. The x-ray suggested the os odontoideum. MRI demonstrated a dramatic compression of the cervical spinal cord and an abnormally high signal intensity area in this region. The patient underwent a midline posterior approach for the treatment of this lesion. Postoperatively, the reduction results were satisfactory and the compression was relieved.

The video can be found here: https://youtu.be/3qDzR2kOz8k.

## Transcript

This video demonstrates a midline posterior approach for the treatment of os odontoideum via atlantoaxial reduction, bone graft fusion, and internal fixation.

This is a 50-year-old man, suffering from four limbs numbness and weakness for about 2 years. And the physical examination found a decreased sensation to pinprick of the skin and an increased muscle tension of the four limbs. Preoperatively, anterior instability was noted on flexion x-ray examination. No instability was noted on extension x-ray. The sagittal CT scan showed a dystopic os odontoideum. The MRI demonstrated that the dramatic compression of the cervical spinal cord and abnormal high signal intensity area in this region.

The patient underwent a posterior approach for the treatment of this lesion via atlantoaxial reduction, bone graft fusion, and internal fixation. The patient was put into a prone position with a Mayfield head frame, and the incision length was about 6 cm along the middle line.

After incision of the skin, the subcutaneous tissue was cut by the unipolar electric knife, strictly along the midline in order to reduce the amount of bleeding. And then we exposed the posterior arch of C1 and the laminar of C2 by subperiosteal dissection. And then we can see the spinous process and vertebral plate of axis. Posterior arch of atlas was isolated meticulously. After exposure of the laminar of C2, we prepared for the screw path and tapping by the freehand and then twisted in a 2.8-cm pedicle screw on the left side of C2. Then the same procedure was performed on the other side.

And then we prepared for the screw path in C1 through arch by using a high-speed grinding drill. It is very safe by using a high-speed grinding drill to make a pedicle screw path in C1. We grasped the posterior arch of C1 using forceps in order to prevent its compression to the spinal cord during the twisting of the screws. The same procedure was performed on the other side. After the four screws were placed in the right place, the lateral x-ray indicated that the C1 and C2 were still in a dislocated position. Then we placed the rod and compressed the screw tails of C1 and C2 little by little, side by side. The lateral x-ray confirmed that the joint between C1 and C2 and the odontoid process were in the right position.

After that, we prepared the bone graft surface by using low-speed drill to grind the surface of the bone graft bed. The allogeneic bone combined with autogenous bone were put onto the surface of the bone graft bed. The bone marrow blood was sprayed at the bone graft bed that was drawn from the iliac bone. At last, we placed a drainage tube and sutured the incision, and finished the operation.

Postoperative CT indicated the satisfactory reduction of C1 and C2 articulus. And postoperative MRI suggested that the compression of the cervical spinal cord was relieved and the signal of the CSF around this area was clear and continuous.

### Time points

0:35 Patient’s history

1:43 Positioning

1:53 Skin incision

2:09 C1 and C2 dissection

2:32 Pedicle screw twist

3:52 Screw and rod tightening

4:04 Bone graft preparation

4:14 Bone graft fusion

4:32 Drainage tube placement and suture of the incision

4:40 Postoperative CT and MRI

5:04 References
